# The roles of gut microbiota and their metabolites in uric acid–related metabolic diseases: mechanisms and therapeutic targets

**DOI:** 10.3389/fmicb.2026.1781413

**Published:** 2026-05-15

**Authors:** Haochen Li, Huijie Zhou, Mingzhen Li, Jinglin Wu, Lei Pan

**Affiliations:** Department of Respiratory and Critical Care Medicine, Binzhou Medical University Hospital, Binzhou, China

**Keywords:** gut microbiota, hyperuricemia, microbial metabolites, purine metabolism, uric acid metabolism

## Abstract

Gut microbiota and their metabolites play a central regulatory role in uric-acid–related metabolic diseases, serving as a crucial interface linking purine metabolism with host inflammatory responses. This review synthesizes current evidence on microbial community alterations, metabolic functional shifts, and receptor-mediated signaling mechanisms in hyperuricemia, gout, and uric acid nephropathy. Studies demonstrate that gut microbes can directly degrade purine substrates, modulate uric acid transporters and metabolic enzyme activity, and regulate host pathways through metabolites such as short-chain fatty acids, bile acids, and indole derivatives acting on receptors including GPR41 and GPR43. These interactions collectively shape the dynamic balance between uric acid production and excretion. Probiotic and prebiotic interventions, such as *Lactobacillus fermentum* GR-3, *Lactobacillus johnsonii* YH1136, and *Lactobacillus gasseri* PA-3, effectively improve uric acid dysregulation via the gut–kidney and gut–liver axes, underscoring their therapeutic potential. In parallel, emerging metabolite- and receptor-targeted strategies provide new opportunities for precision intervention in uric-acid–related metabolic disorders. Future work should emphasize mechanistic causality and individualized host–microbe interactions within the microbe–metabolite–host signaling network to support clinical translation of microbiota-based therapies.

## 1 Introduction

In recent years, the global prevalence of hyperuricaemia (HUA) and related conditions, including gout, urate nephropathy and complications associated with metabolic syndrome, has continued to rise, becoming an urgent public health concern. High levels of uric acid in the blood are strongly linked to an increased risk of gout attacks, as well as to hypertension, chronic kidney disease, cardiovascular disease and type 2 diabetes (El Din et al., 2017; Wang et al., 2025). While uric acid exhibits antioxidant properties at physiological concentrations, excessive levels have pro-oxidative and pro-inflammatory effects (Lanaspa et al., 2012).

**Figure 1:**
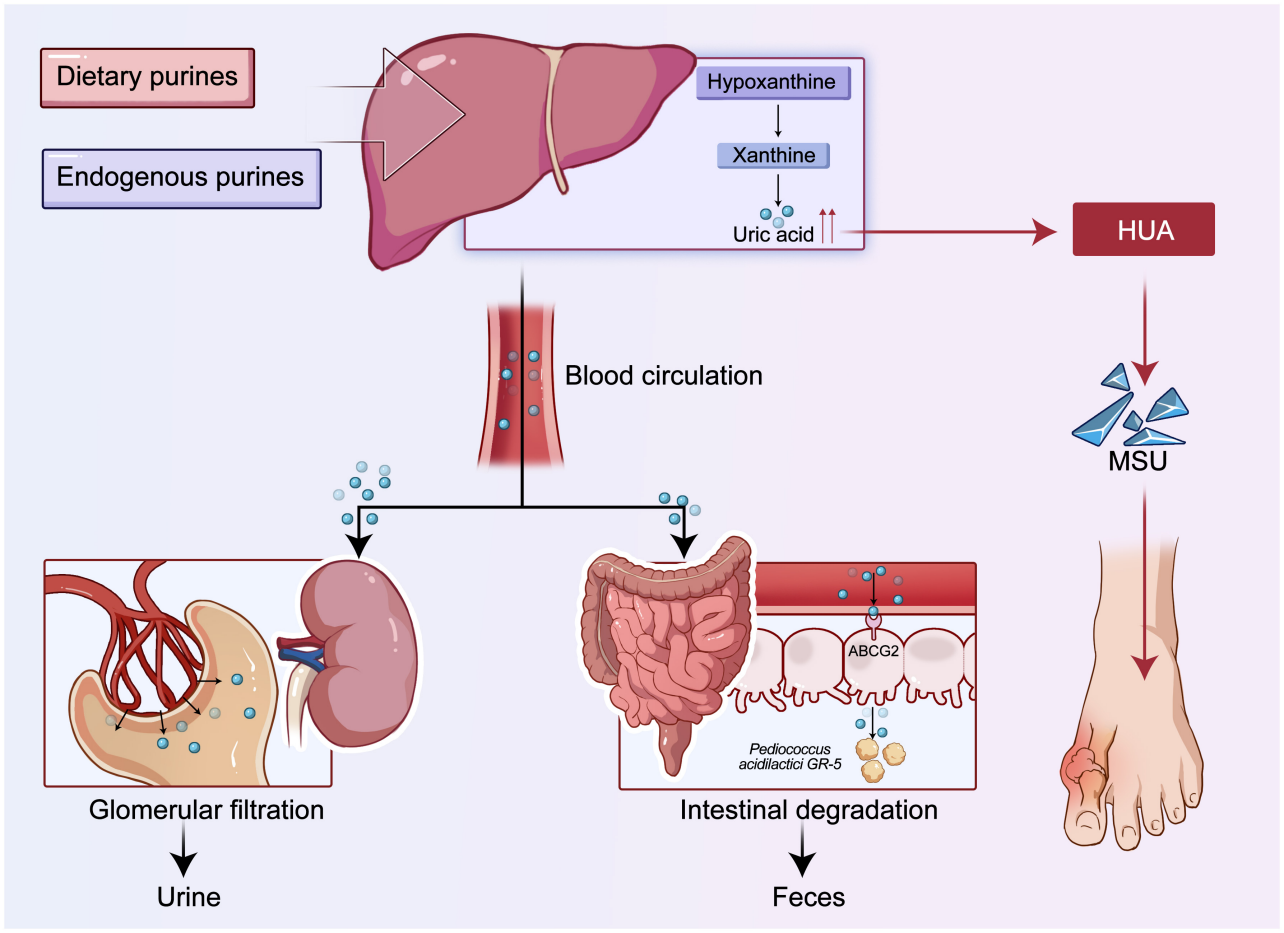
Schematic diagram of purine metabolism, uric acid homeostasis, and renal-intestinal excretion pathways.

At the level of metabolic regulation, urate is the final product of purine catabolism and plays a critical role in its own production and elimination in the human body (Sorensen, 1965). Uric acid is generated through the oxidation of hypoxanthine and xanthine, a process catalyzed by xanthine dehydrogenase (XDH) and xanthine oxidase (XO). These two enzymes constitute the key system responsible for urate formation. Uric acid is primarily excreted and reabsorbed in the proximal convoluted tubules of the kidney and in the intestine. In the kidney, URAT1 (SLC22A12) and GLUT9 (SLC2A9) are the main transporters responsible for reabsorbing serum urate, while transporters such as ABCG2 facilitate uric acid secretion in both the intestine and the kidney (Chung and Kim, 2021; Dai and Lee, 2024). Dysfunction in these pathways can lead to urate accumulation and hyperuricemia, which subsequently promotes the formation and deposition of monosodium urate (MSU) crystals in the joints, triggering gout flares (Chung and Kim, 2021; Liang M. et al., 2025). From a systems physiology perspective, urate homeostasis involves the coordination of purine metabolism in the liver and the regulation of urate transport across multiple tissues, including the kidneys, intestines, and joints. This integrated process constitutes a regulatory network that links signaling pathways, metabolic reactions, and transporter activity in order to maintain urate balance in the body (Sorensen, 1965; Terkeltaub and Dodd, 2025). These core processes of purine metabolism and uric acid homeostasis are illustrated in Figure 1.

The gut microbiota, often referred to as the “second genome,” represents a vast repertoire of microbial genes that coexist with the host genome and has increasingly been recognized as a key contributor to metabolic diseases. Beyond fermenting and metabolizing dietary residues, the gut microbial community participates in multiple biochemical pathways involving amino acids, lipids, and bile acids. Through its influence on host immunity, energy metabolism, and intestinal barrier integrity, the gut microbiota exerts profound effects on metabolic health (Cani et al., 2013; Krautkramer et al., 2021). Recent studies have revealed marked alterations in the gut microbial ecosystem of individuals with HUA and gout, including reduced overall microbial diversity, depletion of short-chain-fatty-acid-producing taxa such as *Faecalibacterium* and *Roseburia*, and enrichment of pro-inflammatory genera such as *Prevotella, Escherichia*/*Shigella*, and members of the *Enterobacteriaceae* family. Concomitant increases in intestinal permeability and impairment of epithelial barrier function have also been reported. These changes suggest that the gut microbiota may influence urate metabolism by modulating purine degradation, intestinal urate excretion, and host inflammatory responses, thereby forming a mechanistic link between dysbiosis and uric-acid-related disorders (Liu et al., 2024; Renton et al., 2025).

Against this background, the present review offers a structured overview of the gut–urate axis. We begin by summarizing current evidence of gut microbial dysbiosis in HUA, gout and related metabolic disorders. We then describe the principal mechanisms through which gut microorganisms and their metabolites influence purine metabolism as well as urate production and excretion. We place a particular emphasis on the multi-layered signaling network underlying microbial-metabolite-host crosstalk, including the regulatory roles of short-chain fatty acids (SCFAs), bile acids, and indole derivatives in modulating urate transporters and inflammatory pathways. Finally, we examine emerging microbiota- and metabolite-targeted intervention strategies, with a focus on translating mechanistic insights into therapeutic opportunities. Our goal is to provide a clearer conceptual framework to support future research and therapeutic development for uric-acid-related metabolic diseases.

## 2 Gut microbiota and uric acid–related metabolic diseases

### 2.1 Clinical evidence of gut dysbiosis in hyperuricaemia

In recent years, numerous population-based studies have consistently shown that HUA and gout are closely associated with gut microbial dysbiosis. The overall patterns include: (i) a marked reduction in α-diversity and a clear separation in β-diversity; (ii) a depletion of butyrate-producing taxa and classical probiotic genera; and (iii) an enrichment of opportunistic pathogens linked to purine–inflammation pathways. Evidence from 16S rRNA sequencing, metagenomics, cross-cohort integrative analyses, machine-learning models and Mendelian randomization studies has demonstrated high reproducibility across different populations and study designs, providing epidemiological support for the “gut microbiota–urate homeostasis–inflammation” framework.

(Chu et al. 2021) conducted a metagenomic analysis involving 307 participants, comprising 102 individuals with gout, 86 healthy controls, and additional individuals in an intermediate state. The gout group exhibited significantly reduced Shannon α-diversity (*p* = 0.0016), markedly divergent gene abundance rarefaction curves (p < 1 × 10^−11^) and clear β-diversity separation based on Bray–Curtis distances. Further functional profiling revealed decreased abundances of genes involved in purine and urate metabolism, as well as the NF-κB inflammatory pathway. This was accompanied by the upregulation of lipopolysaccharide biosynthesis and oxidative stress-related pathways.

(Larsson et al. 2018) reported a higher incidence of urinary tract infections in individuals with gout or hyperuricemia than in healthy controls (p < 0.05), suggesting a link between elevated uric acid, microbial imbalance, and inflammation. Serum uric acid was positively correlated with BMI, although BMI did not differ significantly between the hyperuricemia and healthy groups, indicating that factors beyond obesity contribute to uric acid elevation and gut dysbiosis.

(Tang et al. 2025) integrated 16S rRNA data from five datasets (*n* = 233) and developed an interpretable SHAP-based machine-learning model with 82%−96% accuracy in distinguishing HUA from gout. The analysis identified consistent microbial shifts, with reduced *Faecalibacterium* and *Roseburia* (p < 0.05) and increased *Escherichia* and *Bacteroides* (p < 0.01). The study further proposed an “intestinal ecological gradient” underlying the transition from HUA to gout.

(Martínez-Nava et al. 2025) profiled fecal metatranscriptomes from healthy individuals (*n* = 10), asymptomatic HUA patients (*n* = 10), and gout patients (*n* = 6). They observed strong upregulation of KOs involved in pyruvate, pentose phosphate, and purine metabolism (Log_2_ FoldChange > 22, p_adj_ < 10^−7^), alongside marked downregulation of glycine metabolism and phenylalanine biosynthesis pathways (Log_2_ FoldChange ≈ −18 to −22, p_adj_ < 10^−6^).

In addition to gout and HUA, gut microbial dysbiosis is also closely associated with the formation of urate stones. (Cao et al. 2022) investigated gut microbiota in 117 UAS patients, 123 gout patients and 135 healthy controls. The urate acid stone (UAS) group showed significantly reduced microbial diversity and markedly altered community structures. *Bacteroides* and *Fusobacterium* abundances were positively correlated with serum uric acid (p < 0.05), and *Fusobacteria*-related metabolic upregulation was associated with reduced short-chain fatty acid and amino acid synthesis, indicating a potential metabolic pathway contributing to urate stone formation.

(Ness et al. 2025) analyzed 53 individuals with normal or elevated SUA (eGFR 37–124 mL/min/1.73 m^2^) and found a significant inverse association between Shannon diversity and SUA [β = −36.4, 95% CI (−66.2, −6.7), *p* = 0.017], independent of sex, waist-to-hip ratio and eGFR. Shannon diversity was also positively associated with fractional excretion of uric acid (FEUA) (standardized β = 0.49, *p* < 0.001), explaining 40% of its variance, suggesting that higher microbial diversity may facilitate renal urate clearance.

Multiple systematic reviews have consistently reported that patients with HUA or gout exhibit significant alterations in gut microbiota (GM) community structure, metabolic functions, and ecological network organization (*p* < 0.05). These changes are reproducible across different geographic populations, sequencing platforms, and sample sizes (Singh et al., 2024; Shirvani-Rad et al., 2023).

(Liang et al. 2022) reported that individuals with elevated serum uric acid exhibited significantly reduced α-diversity, as indicated by both the Shannon and Simpson indices (*p* = 0.0029; *p* = 0.013), along with a marked enrichment of *Proteobacteria* (*p* < 0.001) and a reduction in *Ruminococcaceae*_*Ruminococcus* (p = 0.02). This microbial signature-characterized by increased *Proteobacteria* and decreased butyrate-producing taxa-is widely regarded as indicative of impaired gut barrier homeostasis and heightened inflammatory responses, aligning closely with the typical microecological alterations observed under hyperuricemic conditions.

Clinical studies consistently associate HUA with pronounced gut microbial dysbiosis-reduced diversity, enrichment of pro-inflammatory taxa and depletion of butyrate producers-accompanied by functional disturbances in purine and energy metabolism and impaired urate excretion. These findings indicate that microbial disruption is a key feature of HUA.

Current evidence on the gut microbiota-urate axis can be broadly classified into three levels: association exploration, functional validation, and causal inference with mechanistic integration. As summarized in Table 1, this framework reflects the progressive development of research in this field.

**Table 1:** Representative studies across association, functional, and causal evidence levels in gut microbiota research.

Stage	Type of evidence	Key references
1. Association exploration	Reduced diversity, community differences, correlation patterns	(Chu et al., 2021; Cao et al., 2022; Liang et al., 2022)
2. Functional validation	Altered purine metabolism, energy metabolism, and SCFA-related pathways	[Chu (functional annotation); Martínez-Nava et al., 2025; Cao (pathway analysis)]
3. Causal inference and mechanistic integration	Causal direction, metabolic mechanisms, systems-level models	[Ness et al., 2025 (kidney gut axis); Su et al., 2023 (MR); Tang et al., 2025 (ecological gradient)]

In order to objectively present the latest progress in the study of gut microbiota and uric acid metabolism, the following table systematically summarizes high-quality animal experiments and clinical studies published in recent years (Table 2).

**Table 2:** Summary table of studies on the relationship between gut microbiota and hyperuricemia.

Disease / model	Subjects	Study design	Key microbiota findings	Ref.
Hyperuricemia & Gout	Humans (*n* = 368, 4 Chinese cohorts)	16S rRNA sequencing & metagenomic analysis; used ConQuR to correct for batch effects.	Increase in pro-inflammatory bacteria *Fusobacterium* and *Bilophila*; decrease in probiotics Christensenellaceae R-7 and Anaerostipes. Identified a new species SGB Phil1 sp00194085 carrying uric acid metabolism gene clusters.	J Qie et al.
Chronic Hyperuricemia	Mice (PO + Adenine induced)	21-day intervention with Limosilactobacillus reuteri HCS02-001 (Lact-1).	Significantly reduced SUA and Cr; upregulated gut barrier proteins Occludin and Zo-1; inhibited TLR4/MyD88/NF-κB pathway; upregulated ABCG2 expression via the MAPK pathway.	Hussain et al.
Hyperuricemia	Mice (C57BL/6J)	12-week Oleanolic Acid (OA) intervention; validated via Fecal Microbiota Transplantation (FMT).	OA restored microbial balance, increased SCFA production, and strengthened tight junctions. Mice receiving feces from OA-treated donors showed a significant drop in SUA.	Zhang, T. et al.
Intestinal uric acid excretion disorder	Rats (HUA model)	Intervention with Chicory extract, butyrate supplements, and PPAR$\gamma agonists.	Chicory increased butyrate-producing bacteria (*Bifidobacterium, Lactobacillus*); butyrate activated the PPARγ-ABCG2 pathway to promote intestinal uric acid excretion.	Yang, Y et al.
Hyperuricemia	Mice (Kunming)	*L. paracasei* N1115 (high/low dose) vs. Febuxostat control.	SUA reduced by ~29%; downregulated renal URAT1 and GLUT9, upregulated OAT3. Significantly increased Bifidobacterium and intestinal butyrate levels.	Zhang, H. et al.
Gout recurrence prevention	Humans (*n* = 30 with history of gout)	Randomized Controlled Trial (RCT); *L. salivarius* CECT 30632 vs. Allopurinol.	Significant reduction in gout flares in the probiotic group; SUA decreased by ~1 mg/dL. The strain exhibited strong *in vitro* purine metabolism capabilities.	Terkeltaub, R. et al.
Asymptomatic Hyperuricemia (AH)	Humans (*n* = 90, 50% AH)	16S rRNA sequencing and bioinformatic comparative study.	Significant dynamic changes in gut microbial composition in AH patients; certain probiotic proportions showed a compensatory increase in early stages.	Yang, H.T. et al.
Hyperuricemia & colon injury	Mice (Adenine + PO induced)	2-month intervention with *L. rhamnosus* UA260 and *L. plantarum* YU28.	Activated the Aryl Hydrocarbon Receptor (AhR) pathway; repaired the intestinal barrier via tryptophan metabolites (IPA, IAA) and upregulated ABCG2.	Zhuo, Y. et al.

Table 1 compiles 8 studies examining the association between gut microbiota and hyperuricemia, along with its related conditions (including gout, intestinal uric acid excretion disorders, and colon injury). These studies involve both human cohorts and animal models (mice and rats), utilizing diverse research designs such as 16S rRNA sequencing, metagenomic analysis, Randomized Controlled Trials (RCTs), Fecal Microbiota Transplantation (FMT), and targeted interventions. Together, they clarify the central role of gut microbiota in the onset, progression, and management of hyperuricemia.

Regarding gut microbiota composition, patients with hyperuricemia and gout exhibit marked dysbiosis: inflammation-associated bacteria (e.g., *Fusobacterium* and *Bilophila*) are more abundant, whereas beneficial probiotics (such as *Christensenellaceae* R-7 genus and *Anaerostipes*) are reduced. Notably, patients with asymptomatic hyperuricemia already show significant dynamic changes in their gut microbiota, with some probiotics increasing compensatorily in the early stages of the disease. Additionally, a novel species, *SGB Phil1 sp00194085*, which carries uric acid metabolism gene clusters, was identified, offering a new target for investigating disease mechanisms.

Intervention studies demonstrate that several probiotic strains (including *Limosilactobacillus reuteri* HCS02-001, *L. paracasei* N1115, *L. salivarius* (*L. salivarius*) CECT 30632, and *L. rhamnosus* UA260), plant extracts (oleanolic acid and chicory extract), and butyrate supplements effectively lower uric acid levels. Key mechanisms underlying these effects include balancing gut microbiota, enhancing short-chain fatty acid (SCFA) production, repairing the intestinal barrier (via upregulation of tight junction proteins like Occludin and Zo-1), regulating the expression of urate transporters (ABCG2, URAT1, GLUT9, OAT3, etc.), and activating relevant signaling pathways (MAPK, PPARγ, AhR, etc.) or inhibiting inflammatory pathways (TLR4/MyD88/NF-κB). Notably, some probiotics perform as well as commonly used clinical urate-lowering drugs (febuxostat and allopurinol) and effectively prevent gout recurrence.

Collectively, these studies confirm that gut microbiota dysbiosis is a key contributor to hyperuricemia. Interventions targeting gut microbiota structure and function—such as probiotics and plant-derived active ingredients—regulate uric acid metabolism, reduce inflammation, and repair the intestinal barrier through multiple pathways. This provides new insights and potential targets for the safe and effective prevention and treatment of hyperuricemia and its complications, while also laying an experimental basis for the clinical application of microecological preparations like probiotics.

### 2.2 Specific microbial structural and functional signatures

#### 2.2.1 Alterations in the Bacillota/Bacteroidota ratio

In gout and HUA, the gut microbiota frequently exhibits an imbalance between Bacillota and Bacteroidota, most often reflected by a decreased Bacillota/Bacteroidota ratio. Studies conducted in Asian populations consistently report elevated Bacteroidota and reduced Bacillota in affected individuals (Fang et al., 2022; Zou et al., 2024; Demarquoy and Dehmej, 2024).

(Kim et al. 2022) compared gut microbiota across asymptomatic hyperuricemia (asHU, *n* = 8), gout without urate-lowering therapy (0ULT, *n* = 14), short-term urate-lowering therapy (30ULT, *n* = 9) and chronic gout (cULT, *n* = 18). The asHU group showed a significantly higher Bacillota/Bacteroidota ratio than gout groups (p = 0.002), whereas gout patients exhibited reduced Bacillota and increased Bacteroidota, resulting in a lower Bacillota/Bacteroidota ratio. Notably, the Bacillota/Bacteroidota ratio increased after 30 days of urate-lowering therapy, indicating its potential relevance to disease activity and microbial homeostasis. (Renton et al. 2025) summarized metagenomic and 16S studies and described a characteristic “gouty microbiome” marked by reduced α-diversity, an elevated Bacteroidota/Bacillota ratio, and persistent depletion of Akkermansia and Bifidobacterium. These ratio shifts indicate Bacillota loss and Bacteroidota expansion, consistent with heightened inflammation and impaired intestinal metabolic homeostasis. The review highlighted the Bacteroidota/Bacillota ratio as a recurring structural feature of dysbiosis in gout across regions and analytical platforms (Renton et al., 2025).

This imbalance is consistently accompanied by reduced gut microbial diversity. Early metagenomic work further outlined the structural features of gout-associated dysbiosis. In a study of 35 gout patients and 33 healthy controls, (Guo et al. 2016) identified marked species-level and functional differences between the groups. Gout patients showed enrichment of inflammation-related taxa, such as *Bacteroides caccae* and *B. xylanisolvens*, and depletion of butyrate-producing microbes including *Faecalibacterium prausnitzii* and *Bifidobacterium pseudocatenulatum*. Functional analyses indicated disrupted purine degradation and reduced butyrate synthesis, implicating these changes in elevated uric acid burden and mucosal inflammation. A 17-taxon classifier achieved 88.9% diagnostic accuracy, supporting the proposed Microbial Index of Gout as a potential non-invasive marker.

At the genus level, butyrate-producing taxa commonly found in healthy individuals—such as *Faecalibacterium*, Coprococcus, and *Roseburia*—are markedly depleted in patients with HUA or gout, whereas pro-inflammatory genera, including *Prevotella, Bacteroides*, and members of the *Enterobacteriaceae*, are relatively enriched (Table 3) (Yang J. et al., 2025).

**Table 3:** Representative gut microbial taxa associated with gout and hyperuricemia.

Taxa (increased)	Variation	Taxa (decreased)	Variation	Potential impact
*Bacteroides*	↑	*Bifidobacterium*	↓	Inflammation / uric acid metabolism
*Prevotella*	↑	*Faecalibacterium*	↓	SCFA metabolism / barrier function
*Escherichia–Shigella*	↑	*Ruminococcus*	↓	LPS / inflammation
*Fusobacterium*	↑	*Coprococcus*	↓	Pro-inflammatory responses
-	-	*Butyricicoccus*	↓	Butyrate production
-	-	*Akkermansia*	↓	Barrier integrity (reported in some studies)

Overall, gout and HUA exhibit a consistent pattern of gut microbial dysbiosis, characterized by an altered Bacillota/Bacteroidota ratio, reduced diversity, loss of butyrate-producing taxa and enrichment of pro-inflammatory microbes. This signature is widely observed across populations, platforms and disease stages, and is closely linked to disrupted uric acid metabolism, impaired intestinal barrier function and heightened inflammation. Together, these features represent the most stable microbial hallmarks of gout and HUA and provide important clues for mechanistic understanding and microbiota-based therapeutic development.

#### 2.2.2 Microbial functional signatures of purine metabolism in HUA and gout

The gut microbiota helps regulate host uric acid homeostasis through its involvement in purine degradation and salvage pathways, and potentially in related aspects of purine metabolism. Multi-omics studies consistently show that gout and HUA are characterized by disrupted microbial purine metabolism, including upregulated purine oxidation, reduced salvage and catabolic activity, loss of UOX-related functions, and a unidirectional shift in metabolic flux (Cui et al., 2025; Tong et al., 2022).

(Shen et al. 2021) conducted a large-scale serum metabolomics analysis to delineate metabolic abnormalities in individuals with asymptomatic hyperuricemia (asHU) and gout. Untargeted serum metabolomics revealed over 150 altered metabolites distinguishing asHU, gout and healthy controls, with the most prominent changes in purine metabolism-marked elevations in hypoxanthine-, xanthine- and adenosine-related metabolites suggesting enhanced purine oxidation and possibly reduced clearance. Additional disruptions in fatty acid β-oxidation, amino acid metabolism and broader energy pathways reflected increased metabolic stress and inflammatory susceptibility. A metabolite-based model effectively differentiated asHU from gout, suggesting early prodromal signatures. Overall, the results support a metabolic continuum from HUA to gout driven by purine metabolic reprogramming, oxidative stress and impaired energy homeostasis, aligning with microbiome evidence of upregulated microbial purine pathways.

(Asad ul-Haq et al. 2022) used PICRUSt2 to assess microbial functional differences in purine metabolism among gout patients and found that those with uncontrolled uric acid levels after febuxostat treatment showed marked enhancement of predicted purine metabolic pathways. Compared with healthy controls and treatment-controlled patients, the uncontrolled group exhibited higher predicted functional representation of pathways involved in purine biosynthesis, hypoxanthine and xanthine oxidation and nucleotide degradation.

Integrated metagenomic, metabolomic and functional analyses show that HUA and gout share a consistent reprogramming of microbial purine metabolism, marked by enhanced purine oxidation and reduced salvage and catabolic capacity. Serum metabolomics supports this pattern, revealing accumulation of purine oxidation–related metabolites and broader disruptions in energy and amino acid metabolism. This oxidative shift is especially pronounced in patients with poor uric acid control.

### 2.3 Gut microbiota alterations in urate nephropathy and metabolic syndrome

In hyperuricemia-related disorders such as uric acid nephropathy (UAN) and metabolic syndrome (MetS), gut microbial dysbiosis is a central feature of the disrupted gut-kidney-metabolic axis. Patients typically show reduced diversity, loss of key metabolic taxa and enrichment of pro-inflammatory and toxin-producing bacteria. This microecological shift aggravates uric acid dysmetabolism, inflammation, and energy imbalance, potentially contributing to a pathological cascade that accelerates renal and metabolic deterioration (Méndez-Salazar et al., 2021).

Recent clinical evidence emphasizes the liver's role as a primary site for purine metabolism. (Deb and Sakharkar 2021) noted that elevated levels of serum alanine aminotransferase (ALT) and aspartate aminotransferase (AST) show a strong positive correlation with serum uric acid levels (*p* < 0.001), serving as indicators of disturbances in liver energy metabolism and purine metabolic function. (El Din et al. 2017) further summarized the key steps in uric acid production, clarifying that xanthine oxidase (XOD) is the rate limiting enzyme in the purine nucleotide degradation process that oxidizes xanthine to uric acid. This enzyme is mainly highly expressed in the liver and acts as the core node that regulates the balance of host purine metabolism.

Experimental research provides more direct mechanistic evidence. In their study published in the Journal of Biological Chemistry, (Lanaspa et al. 2012) confirmed that high levels of uric acid can induce mitochondrial oxidative stress. By inhibiting the activity of citrate hydratase in the tricarboxylic acid cycle, this leads to increased accumulation of citric acid and lipid synthesis, ultimately resulting in hepatic steatosis and functional impairment. This study reveals the key pathway by which uric acid damages liver cells through oxidative stress under conditions of excessive purine metabolism.

Recent multi-omics evidence shows that hyperuricemia and related kidney injury, including uric acid nephropathy, involve not only impaired renal urate excretion but also systemic disturbances in the gut microbial ecosystem. Data from Chinese and international cohorts indicate that elevated uric acid profoundly reshapes gut microbial structure and function, forming a reinforcing loop linking metabolic syndrome, microbial dysbiosis and disrupted uric acid metabolism.

Using an integrated metabolomic and gut microbiome analysis in a rat model of hyperuricemia-induced kidney injury, (Pan et al. 2020) demonstrated a clear link between urate elevation, renal damage and gut metabolic imbalance. The study identified a distinct amino acid metabolic signature (AUC = 1.00) and parallel microbial shifts marked by enrichment of conditional pathogens and loss of short-chain fatty acid producers such as *Blautia* and *Roseburia*. Functional analyses showed disrupted nitrogen cycling and enhanced urate degradation, indicating that hyperuricemia-related kidney injury reflects combined host metabolic abnormalities and microbiota-driven nitrogen and purine metabolic reprogramming.

(Zhou et al. 2024) established a HUA rat model using UOX gene knockout and demonstrated the causal link between gut microbial imbalance and kidney injury under elevated uric acid conditions. The model displayed renal dysfunction with tubular injury, fibrosis, NLRP3 activation and intestinal barrier damage. HUA-related dysbiosis was linked to increased toxin-producing pathways and renal accumulation of gut-derived uremic toxins. Fecal microbiota transplantation confirmed that HUA microbiota worsened renal and intestinal injury after ischemia–reperfusion, an effect eliminated in NLRP3^−^/^−^ mice, demonstrating an NLRP3-dependent mechanism.

(Liu et al. 2023) compared the gut microbiome and fecal metabolome of early-stage CKD patients with (CKD-H) and without hyperuricemia (CKD-N). Both groups exhibited gut microbial dysbiosis, with CKD-H showing more pronounced shifts, including reduced Bacteroidota, increased *Proteobacteria* and *decreased Ruminococcus gnavus* group. Metabolomics showed broad amino acid pathway suppression in CKD-N, whereas CKD-H displayed enhanced phenylalanine, purine and related metabolic pathways. Multi-omics associations linked these metabolic differences to key taxa, suggesting that gut microbes mediate the metabolic influence of hyperuricemia on CKD progression.

Population studies by (Deb and Sakharkar 2021) showed a strong positive correlation between serum uric acid and liver enzymes ALT and AST (p < 0.001), suggesting an important hepatic regulatory role in uric acid dysmetabolism. This may reflect gut microbiota–driven increases in hepatic oxidative and metabolic stress through altered purine degradation and xanthine oxidase activity. In a large Guangdong cohort (*n* = 6,280), (Duan et al. 2023) further demonstrated that MetS-related gut dysbiosis partially mediates the BMI–SUA relationship, with *Proteobacteria* and *Ralstonia* accounting for 0.94 and 2.76% of the association, forming a characteristic microbial pattern linking obesity, insulin resistance and hyperuricemia.

Overall, evidence shows that uric acid nephropathy and metabolic syndrome share a consistent pattern of gut microbial dysfunction, including reduced diversity, expansion of proteolytic and toxin-producing taxa and depletion of short-chain fatty acid producers. These shifts coincide with widespread upregulation of purine and nitrogen metabolic pathways. This microecological state—marked by *Proteobacteria* proliferation, loss of SCFA-related taxa and intensified purine and toxin metabolism—promotes uric acid accumulation and accelerates kidney injury through increased toxin load, inflammation and disrupted energy metabolism.

## 3 Mechanisms of gut microbiota–mediated uric acid regulation

**Figure 2:**
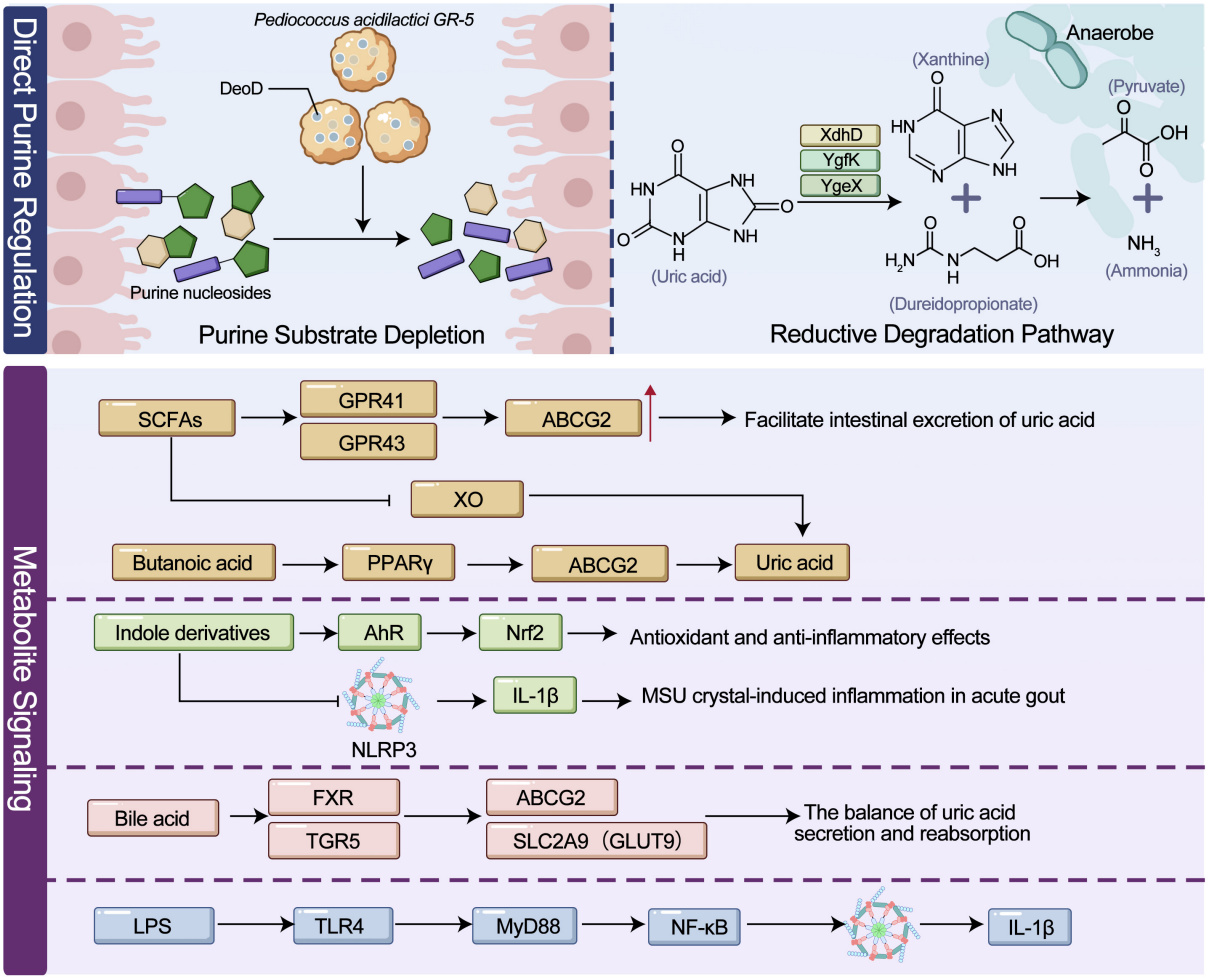
Purine metabolism, uric acid homeostasis, and gout pathogenesis.

Regulation of host uric acid metabolism by the gut microbiota has emerged as a major frontier in research on HUA and gout. Mechanistically, two primary pathways are involved. The first operates through microbial modulation of purine substrate conversion, oxidation and uric acid degradation, thereby directly influencing the balance between uric acid production and clearance. The second involves metabolite-mediated signaling networks, in which short-chain fatty acids, indole derivatives, bile acids and other microbial products regulate hepatic purine metabolism, renal urate excretion and inflammation-related pathways. The interconnected mechanisms of direct substrate modulation andmetabolite-triggered signaling are schematically summarized in Figure 2.

### 3.1 Microbial regulation of purine metabolism

The gut microbiota plays a direct and active role in the host's purine metabolic network. Acting as both a consumer and degrader of purine substrates, it modulates the activity of key metabolic enzymes, thereby influencing the rate and flux of uric acid production (Vogels and Van der Drift, 1976; Kasahara et al., 2023). Recent metagenomic, metabolomic and germ-free animal studies consistently indicate that the gut microbiota is a critical endogenous determinant of uric acid homeostasis (Rodríguez et al., 2023; Han et al., 2021). The kidneys and gut are responsible for approximately two-thirds and one-third, respectively, of total urate elimination (Cui et al., 2025). Gut microbiota can hydrolyse nucleotides, degrade nucleic acids or reuse nucleoside intermediates, forming an ‘intestinal–hepatic purine cycle' that functions alongside the host's own purine metabolism (Kasahara et al., 2023).

Clinical evidence has linked gut microbial alterations to gout. (Shao et al. 2017) revealed significant changes in gut microbiota and metabolic profiles in gout patients through combined fecal metagenomics and metabolomics. Opportunistic pathogens (e.g., *Bacteroides*, Porphyromonadaceae, *Rhodococcus, Erysipelatoclostrium*, and *Anaerolineaceae*) were significantly enriched in patients, while the metabolome exhibited abnormalities related to uric acid excretion, purine metabolism, and inflammatory response. Correlation analysis showed that these characteristic metabolites are closely coupled with specific microbial communities, collectively constituting the complex microbial ecological markers of gout.

Beyond direct microbial composition changes, host–microbe metabolic crosstalk also involves lactate and amino acid metabolism. Through the study of D-lactate dehydrogenase mutations, (Drabkin et al. 2019) reported that enhanced glycolysis leads to increased lactate concentration, which competes with uric acid for renal tubular excretion, thereby inhibiting uric acid excretion and promoting hyperuricemia. (Tang and Chan 2017) showed that branched-chain amino acids (leucine, isoleucine, valine) regulate XOD activity and urea production through the purine nucleotide cycle (PNC), and their metabolic disorders lead to sustained activation of the uric acid production pathway.

A major mechanistic breakthrough came from (Li et al. 2025), who published in *Life Metabolism* the first systematic evidence demonstrating that anaerobic gut bacteria harbor a “reductive uric acid degradation pathway,” independent of the classical oxidative pathway. Researchers identified a complete uric acid degradation gene cluster in *Escherichia coli* that converts uric acid sequentially into 2,8-dioxypurine (xanthine), 2,3-diureidopropionate, and ultimately pyruvate and ammonia. Within this pathway, XdhD catalyzes uric acid reduction, YgfK mediates purine-ring cleavage, and YgeX performs the terminal breakdown reaction. The study also found significantly elevated serum xanthine in gout patients. Introducing this gene cluster into the probiotic *E. coli Nissle* and administering it to uricase-deficient hyperuricemic mice markedly reduced serum uric acid and alleviated renal injury, demonstrating the therapeutic potential of engineered gut bacteria capable of degrading uric acid.

Another well-documented example is (Ji et al. 2025) with *Pediococcus acidilactici* GR-5. This strain harbors a highly active purine nucleoside phosphorylase (DeoD), enabling direct degradation of purine nucleosides in the intestinal lumen. By lowering the absorbable pool of adenosine, hypoxanthine and other purine precursors, it reduces substrate entering host purine pathways and limits uric acid production. In a nucleoside overload-induced hyperuricemia mouse model, GR-5 treatment lowered serum uric acid by approximately 52.17%. Beyond substrate depletion, GR-5 reshapes the gut microbial community, increasing abundance of co-metabolic taxa such as Lactobacillus and enhancing pathways involved in purine, tryptophan and bile acid metabolism. At the same time, GR-5 promotes short-chain fatty acid (SCFA) production, supporting epithelial energy metabolism and maintaining barrier integrity. Thus, GR-5 provides one of the clearest examples of a microbe capable of exerting direct control over host uric acid metabolism.

Based on existing research, the functional differentiation of the main gut microbiota in purine metabolism is shown in Table 4. Different bacterial genera exhibit significant metabolic biases in producing and inhibiting uric acid production. Despite their diverse origins, current studies consistently show that gut microbes can directly modulate host purine supply and uric acid production by degrading purine nucleosides, breaking down uric acid, and altering key metabolic enzyme activities. Both reductive uric acid-degrading anaerobes and nucleoside-phosphorylating functional strains can redirect intestinal purine flux and lower absorbable substrate load. Animal evidence further confirms that this direct microbial regulation effectively alleviates hyperuricemia, supporting microbiota-based targeted interventions.

**Table 4:** Major gut microbial taxa and their functional roles in the regulation of purine metabolism.

Genus / species	Metabolic direction	Primary metabolic function	Key genes / enzymes	Impact on host uric acid levels	Evidence type
*Escherichia coli*	Uric acid–producing	Carries XDH-like gene clusters that enhance purine oxidation	*xdhA/B/C*	↑ Serum uric acid (2.3-fold)	Metagenomics / metabolomics
*Klebsiella pneumoniae*	Uric acid–producing	Enhanced xanthine oxidation activity	XOD, *guaB*	↑ SUA; ↑ gut-derived uric acid flux	Animal model
*Enterococcus faecalis*	Uric acid–producing	High purine nucleotide degradation activity	*purA, guaB*	↑ Serum uric acid	Functional gene prediction
*Lactobacillus reuteri*	Uric acid–lowering	Adenosine degradation and substrate competition	ADA, PNP	↓ Uric acid production rate (35%)	*In vitro* fermentation
*Bifidobacterium longum*	Uric acid–lowering	Inhibits purine nucleotide conversion	PNP	↓ SUA; improved gut metabolism	Cell-based experiment
*Faecalibacterium prausnitzii*	Uric acid–lowering	Converts adenosine/guanosine into SCFAs	*purM, fadA*	↓ SUA (28%); ↓ hepatic XDH (30%)	Germ-free mouse experiment
*Clostridium difficile*	Uric acid–producing	Increased purine degradation activity	*xdhC, purF*	↑ SUA	Metagenomic analysis

### 3.2 Microbial metabolites affecting uric acid production and excretion

Recent multi-omics studies have increasingly revealed that the gut microbiota influences purine metabolism not only through shifts in species composition but also via microbial metabolites that directly modulate host uric acid production and excretion. Among these metabolites, short-chain fatty acids (SCFAs), particularly butyrate and acetate, exert multifaceted regulatory effects. They enhance intestinal epithelial energy supply and barrier integrity, promote the expression of uric acid transporters such as ABCG2, suppress xanthine oxidase activity, and reduce systemic or mucosal inflammation, all of which contribute to lowering uric acid levels.

The role of butyrate in uric acid excretion has been mechanistically linked to the Wnt5a/b–β-catenin signaling pathway and ABCG2 upregulation. In hyperuricemic mice, reduced butyrate levels correlate with impaired uric acid excretion. Interventions that restore butyrate, such as *Lactobacillus johnsonii* supplementation, activate this pathway, enhance ABCG2 expression, and significantly improve intestinal urate transport efficiency (Han et al., 2025). Beyond this specific pathway, SCFAs also serve as intestinal energy substrates and help maintain epithelial barrier integrity, indirectly facilitating uric acid clearance (Han et al., 2025; Wang Q. et al., 2021).

More broadly, the gut microbiota modulates uric acid homeostasis through at least four interconnected mechanisms: promoting intestinal catabolism of purines and uric acid; enhancing intestinal urate excretion via SCFAs; regulating urate transporters including ABCG2; and mediating intestinal inflammatory responses that affect uric acid balance (Wang et al., 2021). In summary, microbial metabolites, particularly butyrate, synergistically regulate uric acid production and excretion by enhancing ABCG2 expression, inhibiting xanthine oxidase activity, and maintaining intestinal mucosal barrier integrity.

### 3.3 Microbe–metabolite–host signaling network

The gut microbiota regulates host purine metabolism, inflammatory responses, and uric acid excretion through a multilevel network, representing a key focus in research on hyperuricemia and gout pathogenesis. Recent studies have established a four-tier mechanistic framework encompassing microbial taxa, microbial metabolites, host receptors, and downstream metabolic responses. Within this framework, metabolites such as short-chain fatty acids (SCFAs), indole derivatives, bile acids (BAs), and lipopolysaccharides (LPS) form a core signaling axis that collectively modulates uric acid production, excretion, and inflammatory processes.

The gut microbiota influences host immunity, lipid metabolism, and intestinal barrier homeostasis through microbial metabolites and their associated signaling pathways, thereby playing a central role in the systemic inflammatory processes observed in hyperuricemia and gout. For instance, (Wang Y. et al. 2021) reported that patients with gout exhibit significantly reduced levels of high-density lipoprotein cholesterol (HDL-C), accompanied by impaired anti-inflammatory capacity. This dysfunction is reflected by the upregulation of pro-inflammatory cytokines and the suppression of anti-inflammatory responses.

At the specific microbial taxon level, (Nii et al. 2023) found that *Prevotella copri* (*P. copri*) strains enriched in the gut of arthritis patients possess high genomic plasticity and can significantly upregulate IL-6, IL-17, and Th17 related inflammatory pathways. (Jiang et al. 2022) further confirmed that *P. copri* promotes macrophage inflammatory response and exacerbates joint inflammation by producing succinic acid and fumaric acid. (Astbury et al. 2020) pointed out that increased abundance of *Collinsella* is closely related to elevated triglycerides and total cholesterol levels and is negatively correlated with HDL. (Liu H. et al. 2022) found in animal experiments that the primary bile acid taurocholic acid can induce distal barrier leakage in the small intestine, suggesting that *Collinsella* mediated bile acid oxidation may participate in inflammation by increasing intestinal permeability.

A consistent pattern emerges from studies on SCFA producing bacteria. (Ni et al. 2021) confirmed that the number of SCFA producing bacteria (such as Lactobacillus and *Faecalibacterium*) significantly decreases in patients with gout and hyperuricemia, while the proportion of toxin producing or inflammatory bacteria increases. This shift leads to weakened xanthine oxidase (XO) inhibitory signals, reduced intestinal barrier function, and sustained activation of host immunity. Collectively, these studies indicate that the gut microbiota interacts with the host through its metabolites (such as SCFAs, succinic acid, and bile acids) and related signaling pathways (such as GPR43, NF-κB, and the Th17 axis), forming a multilevel signaling network.

SCFAs, including acetate, propionate, and butyrate, are primarily produced by members of the Bacillota and Bacteroidota phyla through dietary fiber fermentation. In the gut, SCFAs activate the GPR41 and GPR43 receptors, which subsequently upregulate AMPK and PPAR-γ signaling pathways, thereby improving energy metabolism and enhancing intestinal barrier function (Lee et al., 2024). (Yang Y. et al. 2025) used network biology and animal experiments to confirm the critical role of butyrate in regulating host uric acid excretion. They revealed the mediating mechanism of the “butyrate–PPARγ-ABCG2” signaling axis in promoting uric acid excretion through the gut.

Beyond microbial metabolites, host enzymes also participate in gut–microbe crosstalk. (Crane et al. 2013) found that host XO not only participates in host defense during intestinal infections but also directly affects purine metabolism and uric acid production. During infections with enteropathogenic and Shiga toxin-producing *Escherichia coli*, the intestinal epithelium releases large amounts of XO, leading to a significant increase in local uric acid concentration. XO triggers chloride ion secretion in the intestine, alters epithelial resistance, and inhibits the growth of some commensal bacteria over short periods by oxidizing hypoxanthine to uric acid and producing hydrogen peroxide (H_2_O_2_). However, pathogenic bacteria exhibit high tolerance to this oxidative environment and upregulate Shiga toxin expression using XO induced oxidative signals, thereby enhancing virulence. This study reveals the “metabolic signaling” effect of host XO activity during infection, where purine metabolites and their oxidative intermediates not only participate in antibacterial defense but may also be exploited by pathogens, forming a complex bidirectional regulatory mechanism between microorganisms and host purine metabolism.

**Figure 3:**
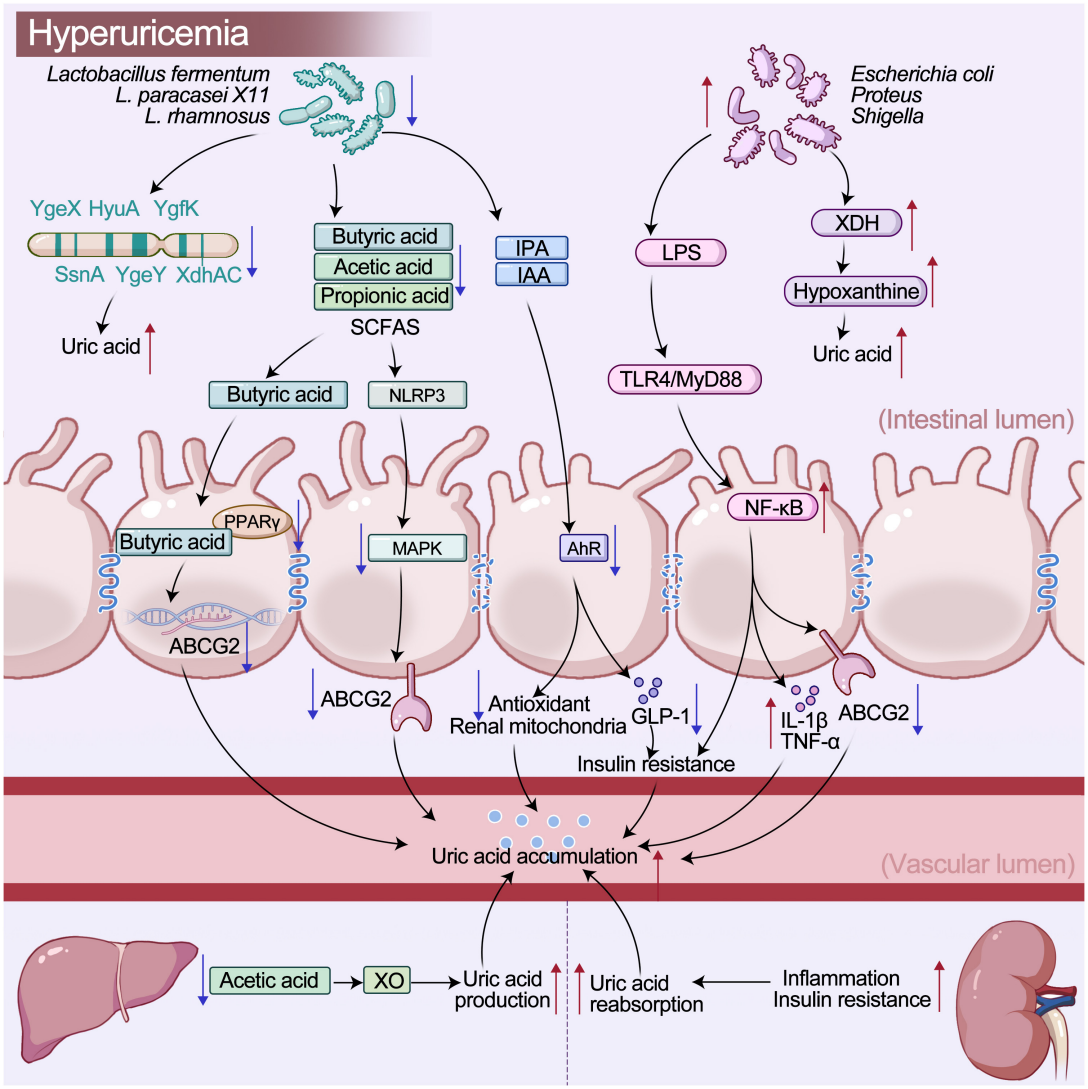
Gut microbiota–metabolite–host signaling network in uric acid regulation.

Based on existing studies, a four-level framework of “microbe–metabolite–host receptor–metabolic response” can be established, as shown in Table 5. Figure 3 illustrates the integrated regulatory network through which gut microbiota and their metabolites control uric acid accumulation in hyperuricemia by modulating intestinal signaling, inflammatory responses, and urate transport. Beneficial taxa such as *Lactobacillus fermentum, L. paracasei* X11, and *L. rhamnosus* act alongside harmful bacteria including *Escherichia coli, Proteus*, and *Shigella*. The highlighted metabolites include SCFAs, IPA, IAA, and LPS. These metabolites modulate pathways involving PPARγ, NLRP3/MAPK, AhR, and TLR4/MyD88/NF-κB, thereby affecting ABCG2 expression, intestinal barrier integrity, inflammation, antioxidant and mitochondrial activity, GLP-1, insulin resistance, and uric acid production, excretion, and reabsorption. Collectively, these processes either alleviate or aggravate uric acid accumulation.

**Table 5:** Four-level mechanistic framework of the gut microbiota–metabolite–host signaling network.

Level	Core components	Representative signaling pathways	Main functional outcomes
1. Metabolite generation	SCFAs, indole derivatives, bile acids, LPS	-	Microbial fermentation, dehydroxylation, and cleavage activities
2. Signal transduction	GPR41/43, AhR, FXR/TGR5, TLR4	GPCR, nuclear receptor, and innate immune signaling pathways	Regulation of energy metabolism, immune responses, and barrier homeostasis
3. Cellular response	AMPK, Nrf2, NF-κB, IL-22	Inflammation- and oxidative stress-related pathways	Modulation of purine oxidation, inflammatory balance, and cellular stress responses
4. Systemic effects	Liver, kidney, and immune system	Purine metabolic enzymes, urate transporters, and cytokine networks	Regulation of uric acid production, excretion, and inflammation

### 3.4 Core mechanisms of gut microbiota in regulating uric acid metabolism

The gut microbiota does not influence uric acid levels through a single pathway but rather constructs a complex regulatory network through multiple levels, including enzymatic degradation, transporter regulation, and inflammatory signaling blockade.

The most direct effect of the microbiota on uric acid levels lies in the biotransformation of purine bases and uric acid within the intestinal lumen. Many probiotics, such as *L. fermentum* and *L. paracasei X11*, can directly hydrolyze purine nucleosides, thereby reducing host absorption of purines (Cui et al., 2025). More importantly, certain specific bacteria contain a complete uric acid degradation gene cluster (including ygeX, hyuA, ygfK, ssnA, ygeY, and xdhAC), and the enzymes encoded by these genes can metabolize uric acid as a nitrogen source (Qie et al., 2025). For example, the newly discovered Phil1 sp00194085 has been confirmed to carry such a gene cluster, providing a biological basis for developing novel uric acid-lowering therapies through engineered strains (Qie et al., 2025).

Conversely, certain pathogenic or opportunistic pathogens, such as *Escherichia coli* and *Proteus*, secrete xanthine dehydrogenase and participate in the oxidative decomposition of purines, which may increase local uric acid concentration under certain conditions (Singh et al., 2024). In addition, *Escherichia* and *Shigella* can secrete xanthine deaminase, converting hypoxanthine into uric acid, further exacerbating the systemic uric acid burden (Singh et al., 2024).

Hyperuricemia is often accompanied by damage to the intestinal barrier. Uric acid and its derived metabolites (such as ammonia) downregulate the expression of tight junction proteins (e.g., Claudin-1, Occludin, ZO-1) (Kim and Song, 2020). When the intestinal barrier is compromised, pro-inflammatory factors such as bacterial lipopolysaccharide (LPS) readily translocate into the bloodstream, triggering systemic low-grade inflammation (Ali, 2025).

This inflammatory state exacerbates metabolism through the following pathways: first, inflammatory cytokines such as IL-1β and TNF-α further inhibit the activity of uric acid transporters in the kidney and intestine via the NF-κB pathway; second, LPS-induced metabolic endotoxemia is a key trigger of insulin resistance (IR), which in turn promotes renal tubular reabsorption of uric acid, thereby forming a vicious cycle (Ali, 2025). Studies have found that intervention with oleanolic acid or specific Lactobacillus strains can significantly repair the intestinal barrier and reduce serum LPS levels, thus disrupting this pathogenic link (Zhang T. et al., 2025).

Uric acid excretion in the intestine mainly depends on transporters on the apical membrane, among which the ATP-binding cassette transporter G2 (ABCG2) is the most critical (Singh et al., 2024). Defects in ABCG2 function have been confirmed to be an important genetic factor in primary hyperuricemia. Nevertheless, the gut microbiota and its metabolites can significantly regulate the expression of ABCG2.

Butyrate, an important microbial metabolite, induces gene transcription of ABCG2 by activating peroxisome proliferator-activated receptor γ (PPARγ) in intestinal epithelial cells (Yang J. et al., 2025). In addition, the MAPK signaling pathway is also involved in the regulation of transporters by microbial metabolites (Hussain et al., 2024). Increasing the abundance of butyrate-producing bacteria (such as *Anaerostipes* and *Collinsella*) can effectively enhance the intestinal “uric acid excretion capacity,” which has extremely important clinical value in patients with renal insufficiency (Qie et al., 2025).

Beyond directly participating in the uric acid cycle, the gut microbiota acts as a “messenger” between the microbiome and host organs by producing a variety of bioactive secondary metabolites.

Short-chain fatty acids (SCFAs) are the main products of dietary fiber fermentation by gut bacteria. In addition to the role of butyrate in regulating transporters, acetate and propionate also show uric acid-lowering potential. Acetate can reduce uric acid production at the source by inhibiting the activity of hepatic xanthine oxidase (XO) (Cui et al., 2025). Moreover, specific probiotic interventions can modulate gut microbiota and increase the production of SCFAs, which is associated with the alleviation of hyperuricemia (Zhang et al., 2025).

In patients with chronic kidney disease (CKD), the loss of SCFA-producing bacteria (such as *Lactobacillaceae* and *Prevotellaceae*) leads to increased intestinal pH and exacerbated inflammation. Supplementation with these bacteria or their metabolites has been shown to delay the progression of renal fibrosis, partly by reducing the systemic uric acid load (Kim and Song, 2020).

Microbial metabolism of tryptophan (Trp) is another important pathway regulating metabolic health. Probiotics such as *Lactobacillus rhamnosus* and *Lactobacillus plantarum* can convert tryptophan into indole derivatives, including indole-3-propionic acid (IPA) and indole-3-acetic acid (IAA) (Zhuo et al., 2026). These molecules are endogenous ligands of the aryl hydrocarbon receptor (AhR).

Activation of AhR is essential for maintaining intestinal homeostasis: Barrier repair: The AhR signaling pathway induces the synthesis of tight junction proteins and reduces LPS translocation (Wang et al., 2024); Metabolic regulation: AhR activation promotes the secretion of glucagon-like peptide-1 (GLP-1) by intestinal L cells, thereby improving glucose tolerance and alleviating obesity and insulin resistance associated with hyperuricemia (Wang et al., 2026); Anti-oxidative stress: In a uric acid nephropathy model, AhR activation helps inhibit oxidative stress in kidney tissue and protect mitochondrial integrity (Liang J. et al., 2025).

The gut microbiota regulates the composition of bile acids (BAs) through dehydroxylation and deconjugation. Bile acids not only participate in fat absorption but also act as signaling molecules to activate the farnesoid X receptor (FXR) and the G protein-coupled bile acid receptor 5 (TGR5) (Ali, 2025). Activation of these receptors regulates host energy metabolism and inflammatory status. Studies have shown that elevated uric acid levels interfere with bile acid metabolism, thereby affecting host lipid homeostasis, which explains why hyperuricemia often coexists with non-alcoholic fatty liver disease (NAFLD) (Ali, 2025).

## 4 Therapeutic targets

The ultimate goal of gut microbiota research is to develop precise diagnostic tools and effective intervention strategies.

**Figure 4:**
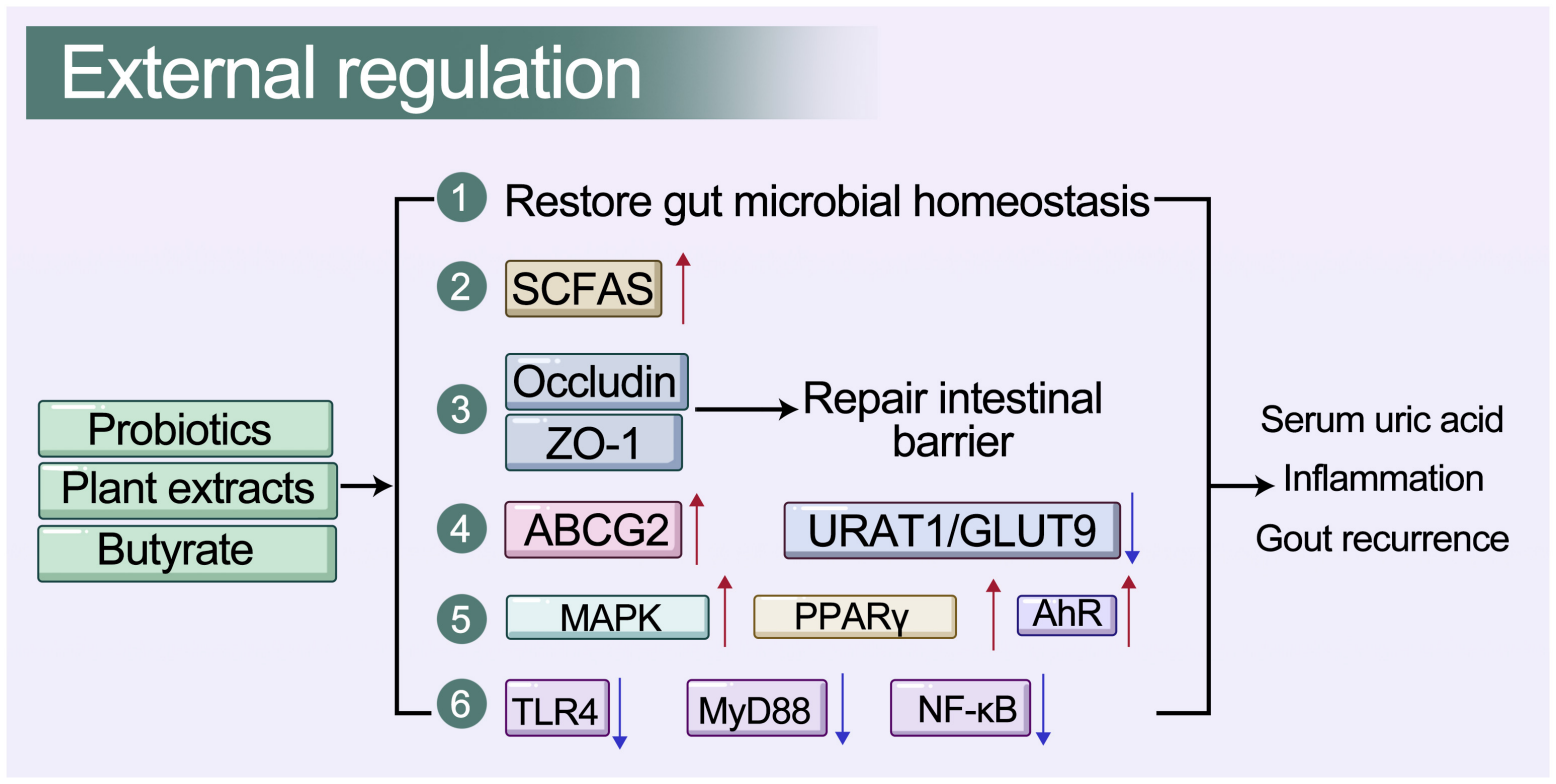
Microbiota-targeted therapeutic strategies for hyperuricemia and gout.

Through analysis of large-scale Chinese cohorts, researchers have identified specific microbial signatures that can distinguish healthy individuals, hyperuricemia patients, and gout patients (Qie et al., 2025). For example, a random forest model constructed using 37 genus-level microbial markers achieved an AUC exceeding 0.8 for discriminating gout from healthy controls (Qie et al., 2025). Furthermore, Mendelian randomization (MR) studies have confirmed causal relationships between certain taxa (e.g., *Ruminococcus* and *Bacteroides*) and gout risk (Liu et al., 2024). These microbial markers hold promise as non-invasive tools for early screening of hyperuricemia. Figure 4 summarizes the primary microbiota-based therapeutic strategies and their underlying mechanisms for managing uric acid–related metabolic diseases.

Current clinical and prospective studies point to the following intervention directions:

Probiotics represent one of the most direct microbiota-targeted strategies. As described above, specific strains such as *L. salivarius* CECT 30632 have demonstrated efficacy in lowering uric acid and reducing gout attacks in randomized controlled trials (Rodríguez et al., 2023). The mechanisms of these live biotherapeutic products include direct consumption of purine precursors in the intestinal lumen and regulation of host physiology through metabolites. Beyond live bacteria, prebiotics and dietary interventions also play a role. Dietary fibers such as chicory and inulin indirectly enhance intestinal uric acid excretion by modulating the proportion of butyrate-producing bacteria (Cui et al., 2025). A high-fiber diet is thought to protect the host from metabolic disorders by maintaining microbial diversity. Another approach, fecal microbiota transplantation (FMT), has been tested in animal experiments: transplantation of fecal microbiota from healthy donors or from donors treated with specific agents significantly improved the hyperuricemic state in recipient mice (Pan et al., 2020). Although not yet widely applied in human gout treatment, FMT holds potential for exploration in severe uric acid stones or nephropathy. Finally, engineered strains and precision enzyme replacement offer a cutting-edge direction. With advances in synthetic biology, scientists are developing engineered gut bacteria that can efficiently express uricase, which may provide a long-term, systemically drug-free supplementation option for patients with hereditary transporter defects (Terkeltaub and Dodd, 2025).

### 4.1 Probiotics and prebiotics

Probiotics and prebiotics, as important means of regulating gut microbiota homeostasis, have been widely used in intervention studies for uric acid metabolism diseases. Numerous experiments and clinical evidence show that specific strains act through multiple pathways, including direct purine/uric acid degradation, modulation of liver XOD and renal transporters, and gut microbiota remodeling with anti-inflammatory effects.

Direct decomposition of purines and uric acid in the gut. (Yamanaka et al. 2019) conducted a randomized double-blind placebo-controlled trial to evaluate the uric acid-lowering effect of *Lactobacillus gasseri* PA-3 yogurt in hyperuricemia and gout patients. Although the full-sample analysis did not show significant differences, in the subgroup with baseline uric acid levels within the SD range of the main population, the PA-3 group showed a significantly greater decrease than the control group (*p* = 0.0378). PA-3 is known for its ability to utilize purine substrates and reduce intestinal purine absorption, supporting the importance of the “gut purine metabolism pathway.” (Zhao et al. 2022) further investigated *Limosilactobacillus fermentum* GR-3 in a randomized double-blind controlled trial with 120 volunteers consuming either GR-3 probiotic yogurt or conventional yogurt. Results showed that probiotic yogurt significantly reduced serum uric acid (26.2% ± 2.3% vs. 8.6% ± 1.1%), enhanced uric acid excretion in urine and feces, and improved the host's anti-inflammatory response. Metabolomics and microbiota analysis further indicated that GR-3 regulates gut microbiota and promotes uric acid metabolism.

Regulation of hepatic XOD activity and renal uric acid transporters. (Zhang X. et al. 2024) investigated the preventive effect of *Lactobacillus johnsonii* YH1136 in hyperuricemic mice. The study found that HUA significantly reduced gut microbiota diversity, especially in the cecum and colon segments. Through 16S rRNA sequencing, the abundance of lactic acid bacteria in the colon was closely related to the model group, indicating their important role in HUA. L. johnsonii YH1136 effectively reduced serum uric acid and significantly improved kidney and liver pathological damage by inhibiting liver XOD activity and promoting renal ABCG2 transporter expression, thereby reducing uric acid accumulation.

Reshaping gut microbiota and alleviating inflammation. (Lin et al. 2024) conducted a randomized double-blind placebo-controlled clinical trial to evaluate three probiotic strains (*Lactobacillus fermentum* TSF331, L. reuteri TSR332, L. plantarum TSP05) in 82 patients with metabolic associated fatty liver disease (MAFLD). After 60 days of supplementation, serum AST, ALT, and uric acid significantly decreased (*P* < 0.05) with no adverse events. Fecal microbiota analysis showed increased proportion of symbiotic microbiota and reduced opportunistic pathogens, especially *Bilophila wadsworthia*. *In vitro* experiments further confirmed that these strains reduce lipid accumulation and inflammatory cytokine expression in HepG2 cells and promote uric acid efflux in Caco-2 cells. (Testorio et al. 2019) investigated prebiotic inulin combined with a low-protein diet in chronic kidney disease (CKD) patients. Results showed that CKD patients had significantly different gut microbiota from healthy controls. A low-protein diet alone increased *Akkermansiaceae* and *Bacteroidaceae* while reducing *Christensenellaceae, Clostridiaceae, Lactobacillus*, and *Pasteurellaceae*. However, when combined with inulin, *Bifidobacteriaceae* significantly increased. The combined intervention group showed significant decreases in serum uric acid (SUA), C-reactive protein (CRP), plasma TNF-α, and NADPH oxidase (NOX2), indicating reduced inflammation. Thus, prebiotics can improve uric acid metabolism and inflammatory status by regulating gut microbiota, providing potential adjuvant strategies for CKD and hyperuricemia-related diseases.

Overall, probiotics intervene in uric acid metabolism through multiple mechanisms: (1) directly decomposing purines and uric acid substrates; (2) regulating liver XOD activity and renal uric acid transporters; (3) reshaping gut microbiota structure, enhancing barrier function, and improving inflammatory status. Meanwhile, prebiotics such as inulin and oligofructose serve as substrates that promote beneficial bacteria and enhance the uric acid-lowering effect. Future research should focus on precise metabolic targets and combined probiotic-prebiotic interventions for hyperuricemia and gout.

### 4.2 Drug development targeting metabolites or receptors

As the regulatory role of gut microbiota in hyperuricemia and gout becomes increasingly clear, new therapeutic strategies targeting microbial metabolites and their receptors have become a research hotspot. Short-chain fatty acids (SCFAs), particularly butyrate, propionate, and acetate, participate in uric acid homeostasis through multiple pathways, including regulating energy metabolism, inhibiting inflammatory pathways, and improving intestinal barrier function. The multi-target effects of SCFAs largely depend on their downstream G protein-coupled receptors GPR41 (FFAR3) and GPR43 (FFAR2), which are key nodes in maintaining intestinal and metabolic homeostasis. Activation of these receptors can influence inflammatory responses, lipid metabolism, and intestinal transporter expression, making them potential directions for developing microbial metabolite mimetics or receptor agonists (Ang and Ding, 2016; Drabkin et al., 2019).

The intestine itself plays a key role in the occurrence and development of hyperuricemia as a major uric acid excretion organ after the kidneys. (Yin et al. 2022) highlighted that the gut maintains uric acid homeostasis through both epithelial uric acid transporters (e.g., ABCG2, SLC2A9) and microbiota-mediated degradation of urate and purine substrates. Thus, therapeutic strategies targeting the intestine—built around microbial metabolites, their receptors, and uric acid transporters—represent a promising new direction for hyperuricemia management.

Among microbial metabolites, SCFAs have attracted particular attention. (Krautkramer et al. 2021) elucidated through a systematic review that gut microbiota metabolites (including SCFAs, bile acid derivatives, indole compounds, and trimethylamine oxide) regulate host energy metabolism, immune response, neural activity, and inflammation. These metabolites are not only co-metabolic end products but also core mediators of signal transmission across multiple boundaries. The authors proposed that combining metabolomics with mechanistic validation can identify microbial metabolic targets for metabolic diseases, including hyperuricemia and gout. The SCFA receptors GPR41 and GPR43 have been implicated in metabolic disorders such as energy imbalance, obesity, diabetes, and chronic inflammation, suggesting their relevance in hyperuricemic conditions (Lee et al., 2024; Cani et al., 2013). Although receptor-targeted therapeutics for hyperuricemia are not yet available, accumulating evidence supports the SCFA–receptor axis in uric acid regulation. For example, butyrate has been shown in animal models to activate the PPARγ-ABCG2 pathway (Recharla et al., 2023; Li et al., 2023), enhance intestinal urate excretion, and consequently reduce serum uric acid. Furthermore, SCFA levels show a significant inverse correlation with serum uric acid, and improvements in intestinal barrier integrity further facilitate urate excretion. These findings collectively suggest that metabolite mimetics, receptor agonists, and microbiota-directed interventions are feasible therapeutic strategies.

Beyond SCFAs, gut-derived microbial extracellular vesicles (GMEVs) have emerged as novel metabolic signaling carriers capable of crossing the intestinal barrier and modulating host metabolic networks. Recent studies indicate that GMEVs exert regulatory effects on inflammation, insulin sensitivity, and hepatic metabolism, highlighting their therapeutic potential (Macia et al., 2020; Taitz et al., 2023). Although their application in hyperuricemia remains nascent, their stability, controllability, and amenability to bioengineering make them a promising next-generation delivery system and therapeutic platform.

In summary, drug development targeting microbial metabolites or their receptors is shifting from simple “microbiota supplementation” toward more precise “signal-level interventions.” Emerging strategies include: (i) SCFA supplements or structural mimetics; (ii) agonists or biased modulators of GPR41/43; (iii) probiotics and metabolically engineered strains that enhance SCFA production; and (iv) GMEV-based delivery systems. By acting on core nodes of the gut–metabolism–urate-excretion axis, these approaches have the potential to overcome the limitations of traditional urate-lowering therapies that rely solely on inhibiting uric acid production or enhancing renal excretion, offering more precise, safer, and mechanistically grounded treatment options for hyperuricemia and gout.

## 5 Summary

Recent advances in gut microbiome research have highlighted that intestinal microorganisms and their metabolic products constitute a key endogenous system regulating host purine–uric acid homeostasis. By directly degrading purine substrates, generating signaling metabolites, and activating multiple host receptor pathways, the gut microbiota modulates uric acid production, excretion, and inflammatory responses at several mechanistic levels. Current evidence shows that gut dysbiosis acts as a pathogenic driver that not only alters the directional flow of purine metabolism but also triggers low-grade chronic inflammation and oxidative stress. This process forms a maladaptive self-reinforcing loop of “microbial imbalance–metabolic disturbance–inflammatory amplification,” which represents a central pathophysiological mechanism of hyperuricemia and gout.

Building on these insights, restoring gut microbial homeostasis and targeting microbial metabolic signaling have emerged as promising therapeutic strategies. Drug development focusing on microbial metabolites and their receptors has begun to take shape. Short-chain fatty acid (SCFA) mimetics and GPR43 agonists, FXR/TGR5 modulators, bile acid derivatives, and indole-3-propionic acid (IPA)–based anti-inflammatory candidates have all demonstrated potential benefits in animal studies. These interventions represent a strategic shift from conventional substrate inhibition toward systemic metabolic reprogramming.

Despite these advances, several challenges remain. The functional output of the microbiota is characterized by high non-linear complexity, where metabolic responses exhibit substantial inter-individual variability driven by the intricate interplay between diets, genetic backgrounds, and environmental conditions. Moreover, most intervention studies are limited to short-term observations, leaving questions regarding long-term microbial remodeling, safety, and durability of therapeutic effects unresolved. Additionally, the extensive crosstalk and multi-target interactions between microbial metabolites and host receptors make precision targeting more challenging. Future research should adopt a multi-omics approach, integrating metagenomics, metabolomics and transcriptomics, to construct comprehensive “microbiota–metabolite–signaling–phenotype” models. These models will be able to identify key regulatory nodes and enable personalized interventions.

Gut microbiota and their metabolites have shifted from being viewed as “associated alterations” to becoming recognized as core pathogenic drivers and therapeutic targets in uric acid–related metabolic diseases. With the continued development of engineered probiotics, metabolite mimetics, and receptor-targeted modulators, multi-layered precision therapies—ranging from ecological reconstruction to signaling rebalance—are poised to redefine the theoretical framework and clinical management of hyperuricemia, gout, and related metabolic disorders.

Furthermore, compared with existing reviews on this topic, the present article provides a more systematic and in-depth summary of the molecular crosstalk within the gut microbiota–urate axis, with an integrated framework spanning microbial metabolites, host signaling pathways, and uric acid transporters. We also highlight the translational potential of novel intervention strategies, including dietary interventions, physical activity, fecal microbiota transplantation, engineered probiotics, and metabolite-targeted therapies. By emphasizing mechanistic connections and clinical translational value, this review offers a more comprehensive and updated perspective that may serve as a valuable reference for future research and therapeutic development in hyperuricemia, gout, and uric acid-related metabolic diseases.
